# JAK3-Mediated Structural Remodeling of β-Catenin Suppresses Epithelial–Mesenchymal Transition: Insights from In Silico Modeling

**DOI:** 10.3390/biology15191738

**Published:** 2026-10-01

**Authors:** Jugal Kishore Das, Jayshree Mishra, Narendra Kumar

**Affiliations:** Department of Pharmaceutical Sciences, Irma Lerma Rangel College of Pharmacy, Texas A&M Health Science Center, Kingsville, TX 78363, USA; jugal.das@snu.edu.in

**Keywords:** Jak 3, β-catenin, adherence junction, epithelial–mesenchymal transitions

## Abstract

Introduction: Janus kinase 3 (JAK3) is a non-receptor tyrosine kinase that phosphorylates multiple proteins, including β-catenin, a critical component of adherens junctions (AJs). We previously demonstrated that JAK3-mediated phosphorylation of β-catenin regulates its basolateral localization and preserves epithelial barrier integrity. However, the structural basis underlying JAK3–β-catenin interactions and their role in epithelial–mesenchymal transition (EMT) remains unclear. Methodology: Here, we employed homology modeling, molecular docking, and structural simulations to investigate phosphorylation-dependent interactions between JAK3 and β-catenin. Results: Our analysis revealed that Src-mediated phosphorylation at Y654 induces conformational remodeling of the β-catenin N-terminal domain (NTD), creating a favorable interface for JAK3 binding and shifting its interaction from the armadillo repeat domain to the NTD. Subsequent JAK3-mediated phosphorylation at Y30, Y64, and Y86 restores β-catenin conformation, stabilizes the E-cadherin/β-catenin complex, and limits nuclear translocation. Phosphomimetic analyses identified distinct structural contributions of these residues. Conclusions: Collectively, these findings reveal a sequential Src–JAK3 phosphorylation mechanism that maintains AJ integrity and suppresses EMT, providing potential therapeutic insights into epithelial cancer progression.

## Simple Summary

Janus kinase 3 (JAK3) is a non-receptor tyrosine kinase that interacts with and phosphorylates several proteins, including β-catenin, a key component of adherens junctions (AJs). We previously demonstrated that JAK3-mediated phosphorylation of β-catenin regulates its localization to the basolateral membrane and is essential for maintaining epithelial barrier integrity. However, the structural basis of JAK3-dependent β-catenin regulation and its implications in epithelial–mesenchymal transition (EMT) remain poorly understood. In the present study, we employed homology modeling, molecular docking, and in silico structural simulations to investigate the molecular interactions between JAK3 and β-catenin and to determine how phosphorylation-induced conformational changes influence AJ stability. Our analyses demonstrate that Src-mediated phosphorylation of β-catenin at Y654 induces significant conformational remodeling of the N-terminal domain (NTD), increasing the spatial separation between critical amino acid residues and disrupting the native protein conformation. These structural alterations create a favorable binding interface for JAK3 by shifting its interaction from the armadillo repeat domain to the NTD of β-catenin. Subsequent JAK3-mediated phosphorylation of Y30, Y64, and Y86 restores the native conformation of β-catenin, stabilizes the E-cadherin/β-catenin complex, and prevents β-catenin nuclear translocation, thereby preserving adherens junction integrity and suppressing EMT. Phosphomimetic substitution analyses further revealed that Y64E and Y86E are functionally neutral, whereas Y30E and Y654E are predicted to have deleterious effects on β-catenin function, underscoring the distinct structural contributions of these tyrosine residues. Collectively, our findings identify a sequential phosphorylation mechanism in which Src primes β-catenin for JAK3 binding, while JAK3 restores its structural integrity to maintain epithelial homeostasis. These results provide novel structural insights into the JAK3–β-catenin signaling axis and suggest that modulation of this pathway may represent a promising therapeutic strategy for preventing EMT and metastatic progression in colorectal and other epithelial cancers.

## 1. Introduction

Janus kinase 3 (JAK3) is a non-receptor tyrosine kinase belonging to the Janus kinase (JAK) family of proteins, which are well recognized for their roles in cytokine-mediated signal transduction and immune regulation through their association with cognate cytokine receptors [1,2,3,4]. In addition to its established immunological functions, JAK3 is expressed in intestinal epithelial cells (IECs), where it plays a critical role in mucosal restitution, epithelial wound healing, and the maintenance of intestinal homeostasis by preventing chronic basal colonic inflammation [5,6,7,8]. We have previously demonstrated that JAK3 promotes mucosal wound repair through cytoskeletal remodeling via its interaction with the actin-binding protein villin [9]. Furthermore, JAK3 expression and its redistribution to the basolateral membrane of IECs are essential for the localization of β-catenin to adherens junctions (AJs), thereby maintaining epithelial barrier integrity and promoting intestinal epithelial function [10]. Adherens junctions (AJs) are dynamic multiprotein complexes that maintain epithelial architecture and tissue integrity. During epithelial–mesenchymal transition (EMT), AJs are progressively disassembled, accompanied by extensive reorganization of the actin cytoskeleton. This process results in the loss of epithelial polarity and cell–cell adhesion, while enhancing cellular migration, invasion, and resistance to apoptosis—hallmark features of cancer progression and metastasis [11]. Within the AJ complex, E-cadherin is a single-pass transmembrane adhesion protein that mediates homophilic cell–cell interactions through its extracellular domain. Its cytoplasmic domain binds β-catenin, which subsequently interacts with α-catenin through its N-terminal region, thereby linking the cadherin complex to the actin cytoskeleton and maintaining the structural integrity of epithelial cell–cell junctions [11]. In the AJ, E-cadherin is a typical single-pass transmembrane protein which participates in homotypic interactions through its ectodomain, interacting with β-catenin through its cytoplasmic domain, which in turn interacts with α-catenin through its N-terminal region [10,12]. Post-translational modifications such as reversible phosphorylation of β-catenin may induce dynamic conformational changes resulting in activation/deactivation and consequently localization of this protein in the AJ [13]. The study of these defining protein–protein interactions in the AJ is complicated by the lack of experimental crystallographic structures of many of these proteins. In the absence of crystal structures of these proteins, homology-based predictive structural modeling provides an elegant and reliable way of not only building a 3D model of these proteins but also predicting their inter- and intramolecular interactions. Molecular dynamic simulation and structural prediction by homology modeling have emerged as fast and efficient tools to analyze structure–function relationships resulting from such post-translational phosphorylation of proteins in several different signaling pathways [14].

The full-length crystal structures of both Jak 3 and β-catenin protein have not been solved, so far. Structurally, Jak 3 is similar to other members of the Janus kinase family of proteins, composed of seven Jak homology domains (JH 1 to JH7), constituting the kinase, pseudokinase, FERM and Sh2 domains, whereas β-catenin has three main regions, namely the unstructured N- and C- terminal tails, which interact with the 12-core conserved Armadillo repeat sub-domains.

We have previously shown the mechanisms and implications of Jak 3’s interaction with β-catenin in AJ [15]. The present study shows the domain-specific interactions of these two proteins via in silico full-length homology modeling and simulation of the structural changes in B-catenin in Jak 3-mediated phosphorylation. Phosphorylation of specific tyrosine residues on β-catenin and the consequent conformational changes in the protein are not only the key determinants in defining the plasticity of AJ but also determine the fate of β-catenin in the AJ. Earlier studies have provided theoretical insights into the role of β-Catenin in the canonical Wnt signaling pathway by integrating mechanistic modeling and simulation [16]; however, similar studies analyzing post-translational structural modifications in β-Catenin and its functional implications in the regulation of AJ are lacking.

## 2. Materials and Methods

### 2.1. In Silico Homology Modeling of Full-Length Jak3 and Its Structural Components

Jak3 tyrosine protein kinase has seven defined regions of homology known as Janus homology (JH) domains. Although the crystal structure of JH1, the catalytic domain of Jak3 protein, has been solved (pdb id: 1Yvj), the crystal structures of both the full-length Jak3 protein and its other domains, such as FERM, Sh2 or FERM-Sh2 domain, have not yet been solved. The similarity and alignment of the template sequence (preferably with an available crystal structure) with the query are essential for a successfully computationally modeled structure. The crystal structure of the FERM-SH2 domain of Jak2 protein has been solved (8). The FERM domain of Jak3 was modeled using the intensive modeling mode of the Phyre2 web-based server (9), and the crystal structure of the FERM domain of Jak2 was used as the template for annotation of the protein. The Sh2 domain and the FERM-Sh2 domain, complexed together, were also modeled separately by also using the Phyre2 homology-modeling software, to determine their role in the binding of Jak3 to β-catenin protein. Phyre2 is a web-based protein modeling software used to predict and analyze protein structure, function and mutations. The intensive modeling mode of the Phyre2 server can combine multiple template modeling and simplify ab initio protein folding simulations to create the full-length model of the protein. The modeled structures were visualized, annotated and simulated in Accelerys Discovery Studios (Dassault Systems, Biovia, San Diego, USA) and validated by constructing the Ramachandran plot. The full-length Jak3 sequence (UniProt P52333) was submitted to Phyre2 in intensive mode, which assembles a single model from the set of templates detected by HHsearch v3.0.3 and rebuilds template-free segments by ab initio loop modeling. The solved Jak3 kinase (JH1) domain (pdb id 1Yvj) and the crystal structure of the Jak2 FERM–SH2 module were the principal templates; the Phyre2 confidence score and percentage query coverage returned for the full-length model and for each separately modeled domain are reported in Section 3.1. Phyre2 was used in preference to more recent end-to-end predictors because it allowed explicit control of template selection, reported per-region confidence and coverage for the template-free N-terminal segment, and allowed the phosphomimetic variants to be rebuilt and energy-minimized within the same CHARMm framework used for all subsequent docking. Model reliability was assessed against experimental data rather than against a second predictor, by Ramachandran analysis and by superimposition of the modeled JH1 domain on its crystal structure.

### 2.2. Homology Modeling and Phospomimetic Simulations of β-Catenin Protein

β-catenin is a pleotropic transcriptional activator with multiple roles in maintenance of the epithelial barrier function in addition to its role in the Wnt/ β-catenin signaling pathway [17]. The crystal structure of β-catenin has been solved (pdb id 2z6g), but without the unstable extension of the NTD, which contains the tyrosine residues shown in our earlier work to be phosphorylated by Jak3 (10). The full-length protein structure of β-catenin was modeled using Phyre2 and the phospomimetic counterparts were modeled and simulated using ab initio protein folding simulations and the CHARMM forcefield in Phyre2 and Accelyrys Discovery Studios. The modeled structure was comprehensively validated by not only constructing the Ramchandran plot but also superimposing the modeled structure on the crystal structure of the protein. Phosphomimetic variants were generated by substituting the relevant tyrosine residues with glutamate in the validated full-length wild-type model and re-minimizing the structure with the CHARMm forcefield using identical parameters in every case. The variants modeled were the single substitutions Y30E, Y64E, Y86E and Y654E, the double substitution Y30/654E, and the multi-site substitution Y30/64/86/654E. Each variant was superimposed on the wild-type model, which served as the reference structure throughout, so that the conformational effect of each individual substitution could be separated from the combined effect of several, and stereochemical quality was re-checked by Ramachandran analysis before any model was used for docking.

### 2.3. In Silico Protein–Protein Docking Analysis of Y654E β-Catenin and Jak3

Prior phosphorylation of β-Catenin at Y654 is a pre-requisite for further phosphorylation of the same protein in its NTD (Y30, 64, 86) by Jak3. In order to assess the impact of structural changes in β-Catenin on phosphorylation of Y654, which facilitates its interaction with Jak3, we docked and compared the phospomimetic variant of β-Catenin (Y654E) to its wt β-Catenin counterpart using GRAMMx Protein–Protein Docking Web Server v.1.2.0 [18]. The Global Range Molecular Matching (GRAMM) methodology approach was used for smoothing the intermolecular energy function by changing the range of the atom–atom potentials. The methodology also locates the area of the global minimum of intermolecular energy for structures of different accuracy by using Fast Fourier Transformation (FFT) for global search for best rigid body conformation. The hydrogen-bond and other protein–protein interactions were analyzed using Accelyrys Discovery Studios (Dassault Systems, Biovia, San Diego, CA, USA) Table 1. The web-based GRAMMx docking server is the optimum choice for protein–protein docking and was utilized in our study for all the docking analyses. GRAMMx returns a ranked list of candidate complexes. For every run, the ten top-scoring solutions were retained and inspected, and the rank-1 (highest-scoring) solution was carried forward for all hydrogen-bond, interface and superimposition analyses reported here. Wild-type and phosphomimetic models were docked independently rather than by transferring a pose from one to the other, and every run used the same receptor–ligand assignment (β-Catenin as receptor and Jak3 or its isolated domains as ligand), the same default server parameters and no interface restraints, so that wild-type and variant complexes are directly comparable. The structures used were the full-length Phyre2 models of wild-type and phosphomimetic β-Catenin described above, the full-length Phyre2 model of Jak3 together with its separately modeled FERM, Sh2 and FERM-Sh2 domains, the crystal structure of the Jak3 kinase domain (pdb id 1Yvj) and E-Cadherin (pdb id 1q1P). Structural deviations were quantified in Accelrys Discovery Studios by superimposing each variant model, or each docked complex, on the corresponding wild-type structure, which was used as the reference in every comparison; superimposition was carried out over the complete structure rather than over the interface alone, so the values reported reflect global reorganization, including rigid-body reorientation of one partner relative to the other, rather than local backbone deviation within a single domain. Predicted interfaces were then assessed for consistency with previously reported experimental data, in particular our earlier identification of Y30, Y64 and Y86 as the Jak3 phosphorylation sites in β-Catenin and the experimentally defined E-Cadherin-binding surface formed by the armadillo repeats.

### 2.4. Docking Analysis of JH1, FERM, Sh2 and FERM-Sh2 Domains of Jak3 with β-Catenin

An in-depth structural analysis of Jak3–β-Catenin association was studied by individually modeling the domains of Jak3 and docking these domains with β-Catenin protein. As mentioned earlier, FERM and FERM-Sh2 domains were modeled separately using Phyre2 homology modeling software. The domains were docked with β-Catenin using the GRAMMx docking server and the interactions were analyzed for hydrogen bonding and RMSD values using Accelyrys Discovery Studios visualization software 2017 R2 (Accelrys Software Inc., San Diego, CA, USA).

### 2.5. Docking Analysis of β-Catenin with E-Cadherin Protein

Prior phosphorylation of β-Catenin at Y654 induces conformational changes in its structure, which enables it to leave the AJ due to the reduced affinity of its interaction with α-Catenin and E-Cadherin protein. We have previously shown that β-catenin’s association with α-catenin is reduced during phosphorylation of Y654, but the association is restored upon further phosphorylation of Y30,64,86 in the NTD of β-catenin by Jak3. We analyzed the structural implications of phosphorylation of Y30,64,86 by performing comparative docking analysis of Y654E Beta-Catenin, wt Beta-Catenin and Y30,64,86E Beta-Catenin with E-Cadherin protein (pdb id:1q1P). The GRAMMx Protein–Protein Docking server and Accelerys Discovery studios were used for visualization and analysis of the comparative docking. The E-Cadherin coordinates used (pdb id 1q1P) correspond to the ectodomain fragment comprising modules EC1–EC2. Because the experimentally established β-Catenin-binding site of E-Cadherin lies in its cytoplasmic tail, this comparative docking is reported as a relative index of the conformational change produced in β-Catenin by phosphomimetic substitution and not as a model of the native adherens junction complex; this constraint is stated explicitly in the Discussion. In addition, glutamate reproduces the negative charge of a phosphotyrosine but not its size, its geometry or the dianionic character of the phosphate group at physiological pH, and it cannot recapitulate phosphate-specific hydrogen bonding or recognition by phosphotyrosine-binding modules. The substitutions used here therefore report the electrostatic and steric consequences of the amino-acid change rather than of phosphorylation itself, and all comparisons between wild-type and variant models are correspondingly qualitative.

## 3. Results

### 3.1. Structural Determination and Molecular Simulation of Full-Length Jak3 and Structural Determinants of Jak 3

**Figure 1:**
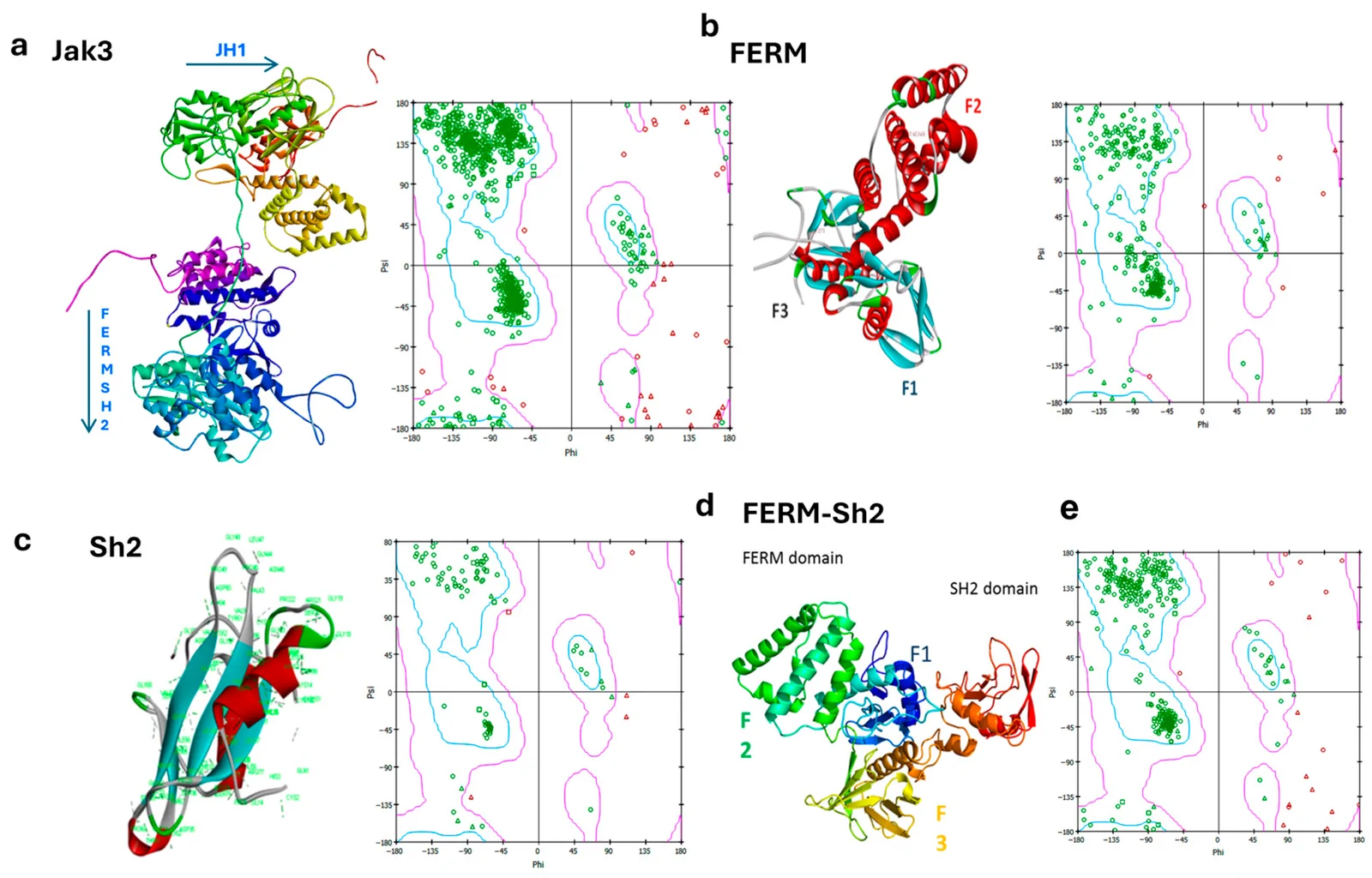
Homology modeling of JAK 3, FERM and FERM-Sh2: Homology modeling of full-length JAK 3 protein was done using the intensive modeling mode of Phyre2 server. A: Stereo ribbon diagram of the protein complex is shown in the figure. JAK3 contains the FERM domain belonging to the ezrin–radixin–moesin (ERM) family of proteins. The SH2 domain is complexed with the FERM domain and plays a central role in autophosphorylation as well as deactivation of JAK 3. The kinase (JH1) domain is classically implicated in JAK 3-mediated phosphorylation of proteins, whereas the pseudokinase domain (JH2) possibly has a structural role in maintaining JAK 3 activity. B: The full-length crystal structure of FERM domain has not been solved for JAK 3, so far. We modeled the FERM domain structure by using Phyre2 homology modeling software. The tri-lobed clover domain structure contains F1, F2 and F3 lobes. Amino acid residues 26-107 constitute the first sub-domain, F1. F2 sub-domain has an Acyl-CoA binding fold and is entirely helical (residues 145 to 271). The third sub-domain, F3, resembles the plekstrin-homology domain (PH) and contains two large, disordered loops, β1–β2 and β3–β4. C: The Sh2 domain is associated with the F3 region of the FERM domain by linker L3. The homology model for the Sh2 domain of JAK3 protein was also constructed by using the intensive modeling mode of the Phyre2 server. D: The FERM and Sh2 domains are complexed in JAK3 protein and regulate the activity of the protein in complex (ref). E: The Ramchandran plot validates our homology model of the FERM and Sh2 domains of JAK3 protein, as all the residues lie in the allowed region of the plot. Panel positions: (**a**), (**top left**); (**b**), **top right**; (**c**), **bottom left**; (**d**), **bottom right**. The Ramachandran plot validating each model (**e**) is shown immediately to the right of the corresponding model. These panels show Ramachandran plots associated with structural models. The colors appear to represent the conformational regions and residue positions: Green circles/dots: residues falling within the allowed/favored conformational regions. Blue contour: most favored φ/ψ-angle region, Magenta contour: allowed conformational region extending beyond the favored region while Red triangles/markers: residues located outside the favored/allowed regions (outliers).

We have previously shown that the interaction of full-length Jak3 with β-catenin facilitates the formation of AJ and increases colonic barrier function. Specifically, Jak3 phosphorylates β-catenin protein at Y_30,64,86,_ after its phosphorylation at Y_654_ by Src kinase. To study protein folding dynamics in the interaction of Jak3 and β-catenin, we constructed a homology model of full-length Jak3. The homology model was constructed by Phyre2 homology modeling software (Figure 1a). The structure was further validated by the construction of the Ramchandran plot in SAVES server (5.0), which shows that ~95% of amino-acid residues lie within the allowed region of the plot. The predicted three-dimensional structures of full-length JAK3 and the separately modeled FERM, SH2, and FERM–SH2 domains are shown in Figure 1. The stereochemical quality of the predicted models was assessed using Ramachandran plot analysis. The corresponding plots are presented alongside each structural model and provide a visual assessment of the backbone dihedral-angle distributions of the modeled residues. Overall, the models exhibited acceptable stereochemical characteristics based on the observed Ramachandran plots. The conserved domains of Jak3 include the carboxy-terminal kinase domain (JH1 domain), the pseudo-kinase domain, an amino-terminal FERM domain (Band 4.1, Ezrin, Radixin, Moesin homology domain) and src homology 2 (Sh2)-like region [19]. These domains are the structural determinants of the full-length Jak3 protein, which modulates its interaction with other proteins like β-catenin. Among the different domains of Jak3, the crystal structure of the carboxy terminal kinase domain of the protein has been solved (pdb id 1yvj), but the FERM and SH2 domains have not been experimentally crystallized, so far. To analyze the domain-specific interaction of Jak 3 with β-catenin, we constructed homology models of all its domains separately. The FERM domain has been shown to be significant in binding to the associated proteins of Jak3 [9]. The in silico-modeled structure of the Jak3 FERM domain was constructed from its analogy to its template in the FERM domain of Jak2 protein (Figure 1b). Secondary structural elements of the model structure of the FERM domain were characterized by superimposition with the crystal structure of the Jak2 FERM domain. We found that the characteristic three-lobed architecture of the FERM domain [20,21] is retained in Jak3, composed of an F1 lobe containing a ubiquitin-like fold, an F2 lobe consisting of an acyl-CoA binding protein fold, and an F3 lobe consisting of a pleckstrin-homology (PH) fold (Figure 1b). Amino acid residues 26–107 constitute the first sub-domain, classified as F1, of FERM. Similarly, the F2 sub domain is composed of residues 145–271 and is entirely helical in structure. The third sub-domain, F3, resembles the plekstrin-homology domain (PH) and contains two large, disordered loops, β1–β2 and β3–β4. The F3 lobe is connected to the Sh2 domain by linker L3, whereas the F1 lobe is connected to both the kinase and pseudokinase domains. Previously our group reported that the phosphorylation of the Sh2 domain was necessary and sufficient for the interaction of cytoskeletal protein villin with the FERM domain of Jak 3 [5,9]. Further, to decipher the interaction of FERM-Sh2 with the β-catenin protein in the AJ, we determined the 3D homology-modeled structure of not only the FERM domain but also Sh2 and FERM-Sh2 complexed together. The intramolecular hydrogen bonds and the amino-acid residues of the Sh2 domain are shown in Figure 1c, and the FERM-Sh2 domain complex is shown in Figure 1d. The Ramchandran plot constructed for the FERM-Sh2 domain complex shows that 98% of the residues lie within the allowed region (Figure 1e) and the model is suitable for further docking analysis.

### 3.2. Refinement of Crystal Structure of β-Catenin and Its Post-Translational Modifications

**Figure 2:**
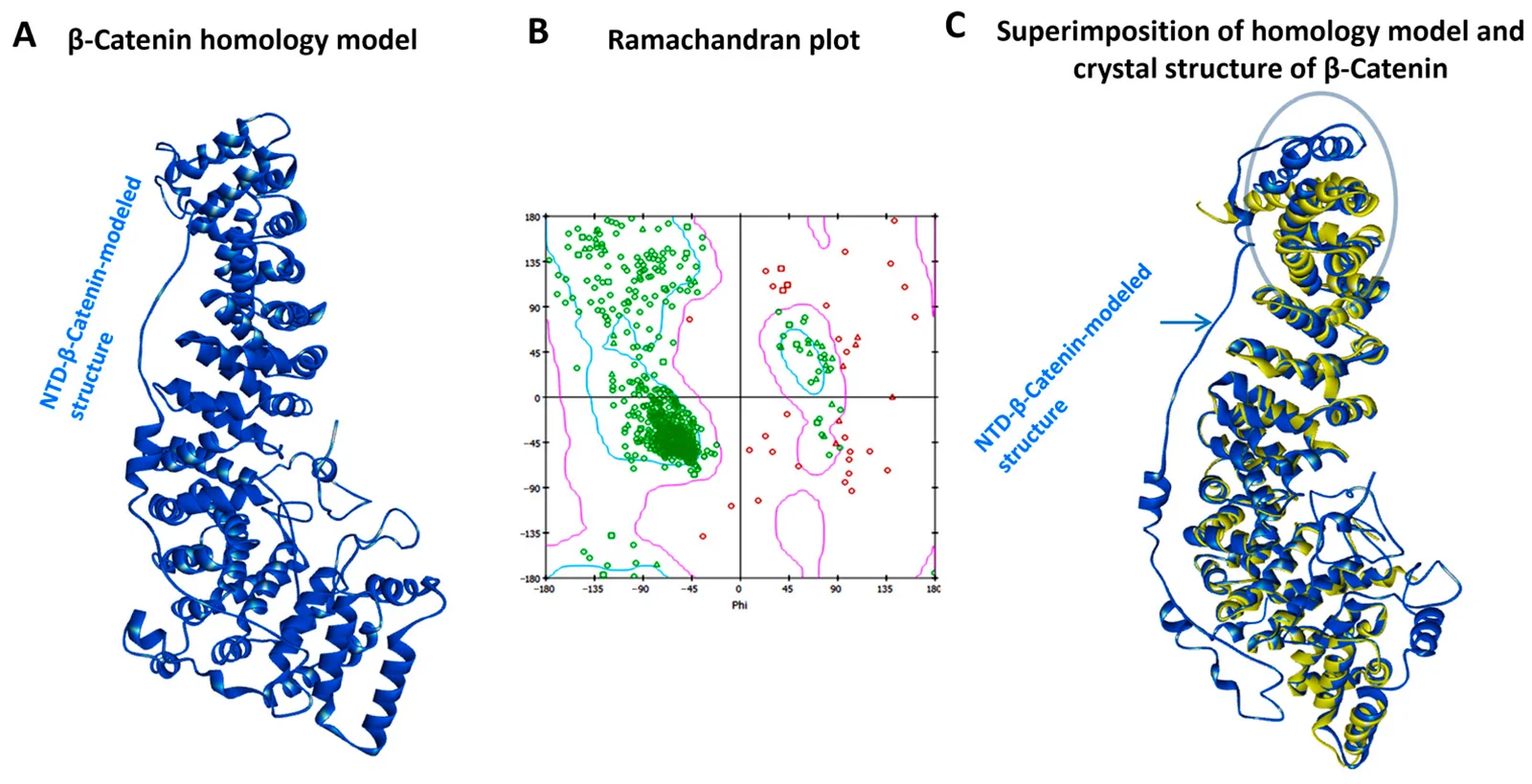
Homology modeling of β-Catenin and superimposition: Although the full-length protein structure of β-catenin is available, the structure does not contain the first 150 amino acid residues of the protein. (**A**). Since the tyrosine residues Y30, 64, 86 lie in the unsolved region of the protein structure, we modeled the full length from its crystal structure using the Phyre2 molecular modeling suite. The structure shows the energy-minimized full-length protein. (**B**). The Ramachandran plot constructed for the model structure validated the in-silico model of the protein. (**C**). The model structure (represented in white) was also superimposed on the β-Catenin full-length protein structure (shown in red). The perfect superimpositions of the protein structures validated the structure while also indicating the structure is amenable for further docking analysis. Panel positions: (**A**), **left**; (**B**), **center**; (**C**), **right**. Figure 2 contains panels (**A**–**C**) only; the conformational effect of the Y654E substitution is shown in Figure 3B.

**Figure 3:**
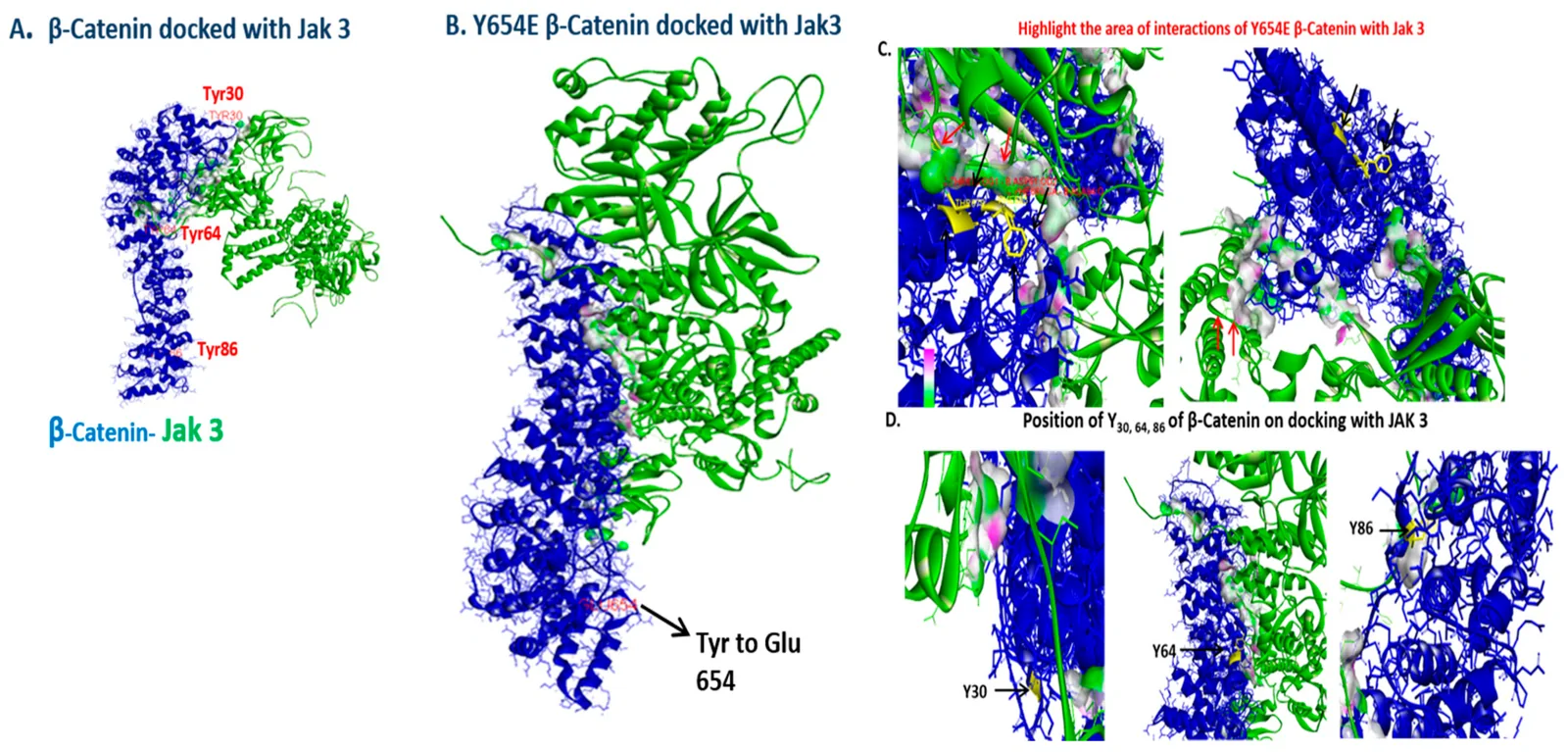
Docking of JAK 3 with β-Catenin: (**A**) GRAMM-x protein–protein docking server was used for docking JAK3 full-length protein with β-Catenin. In the wild-type model the armadillo repeat region of β-Catenin binds the FERM domain of JAK 3. The hydrogen bonds show the contact points of β-catenin shifting to NTD of the protein on phosphorylation of Y30. (**B**) A magnified version of the interactions is shown in the figure. The phospomimetic conversion of Tyrosine to Glutamic acid (Y30E) induces conformational changes in the protein, shifting the binding of JAK 3 to NTD of β-Catenin. The Y64 and Y86 residues are shown in the figure. The Y64 and Y86 residues lie within the hydrophobic region of interaction of the JAK3 and β-Catenin. (**C**) The ligand–protein interactions of JAK3-β-catenin are simulated, and the energy is minimized for the complex using CHARMm forcefield. Red arrows indicate predicted residue-residue contacts between Y654E β-catenin and JAK3 at the docking interface. Panel (**D**) shows the hydrogen-bond monitor output for the docked complex in both receptor–ligand orientations, together with the corresponding magnified views of the interface; the interacting residues are listed in Table 1.

β-catenin plays a pivotal role in maintaining the stability of AJ via modulation of E-cadherin-mediated adhesion and Wnt/β-catenin signaling [16,22]. In our previous study, we have shown that in differentiated human colonic epithelial cells, Jak3 redistributed to basolateral surfaces and interacted with adherence junction (AJ) protein β-catenin. Jak3 expression in these cells was essential for AJ localization of β-catenin and maintenance of epithelial barrier functions. Jak3 is essential in the colon, where it facilitates mucosal differentiation by promoting the expression of differentiation markers and enhanced colonic barrier functions through AJ localization of β-catenin. The dysregulation of this network may lead to epithelial–mesenchymal transition [23]. Post-translational modifications in β-catenin, including phosphorylation, can lead to structural changes regulating the interaction and association of this protein with its partners in AJ. In another study, we have reported that Jak 3 phosphorylates β-catenin at specific tyrosine residues (Y_30, 64, 86_) which determine the fate of β-catenin in the AJ. To study the detailed structural modification induced by phosphorylation of β-catenin, we constructed the full-length model structure of β-catenin (Figure 2A). The structure was further validated and verified by using the Ramchandran plot, which shows the majority of residues lying within the allowed region of the plot (Figure 2B). The superimposition of the homology-modeled in silico structure with the crystal structure of the protein (pdb id 2Z6g) reveals that the modeled structure has an unstable extension of NTD, which is not present in the crystal structure (Figure 2C). Major conformational changes such as in the NTD were observed upon substitution of Y654E, as shown in Figure 3B, in which the Y654E substitution is indicated on the structure; Figure 2 itself contains panels A–C only. Each phosphomimetic model was additionally built and superimposed on the wild-type structure as a single substitution before the multi-site models were assembled, so that the conformational change attributable to Y654E alone could be distinguished from the combined effect of several substitutions. Src-mediated prior phosphorylation of this residue is essential for the interaction of β-catenin with Jak 3. The Y654E-induced rearrangement lies within the region of β-catenin that carries the α-catenin-binding surface; because the β-catenin–α-catenin complex was not modeled in this study, a consequent effect on α-catenin binding remains a prediction to be tested experimentally rather than a result established by the present analysis. However, Jak3-mediated phosphorylation of Y 30, 64 and 86 preserved the native structure of β-catenin, although shifting the binding site for Jak3 towards the NTD.

### 3.3. Molecular Association of Full-Length Jak 3 Protein with β-Catenin and Its Phosphomutant Structure

Jak3 protein interacts with β-catenin and phosphorylates it, inducing structural changes in the protein. To analyze the interaction of Jak3 with β-catenin, an in silico docking strategy was adopted using GRAMMx Protein–Protein Docking server. GRAMMx uses an FFT-based approach to determine the best surface match between molecules. A significant feature of this software is that it accounts for possible conformational changes in protein structures by smoothing surface representations within a rigid body docking approach. In our full-length protein docking analysis, the FERM domain of Jak3 docked onto the armadillo repeat region of wild-type β-catenin (Figure 3A). A single amino-acid phospomimetic substitution of Y30E induces conformational changes in NTD of the protein, which shifts the binding site from the ARM region of β-catenin to the NTD. The simulated docking studies show that Y64 and Y86, which were shown to be crucial in determining the binding of β-Catenin to Jak3 [24], lie within the hydrophobic core region of the interactions (Figure 3B) between the two proteins. A magnified view of the interaction between the amino acids is represented in Figure 3C. Besides Y64 and Y86, the predicted interface is stabilized by hydrogen bonds involving Lys14, Arg57, Arg329, Gly392, Gly393, Ser497, Thr679, Arg684 and Tyr724, by salt-bridge-type contacts with Glu620, Glu1041, Glu1069, Asp91 and Asp755, and by hydrophobic contacts contributed by Met1, Phe34, Ala94 and Ala468; the hydrogen bonds identified in both receptor–ligand orientations are listed in Table 1 and shown in Figure 3C, and those formed between Y654E β-catenin and Jak3 are given in Table 2. Wild-type and Y30E β-catenin were docked independently under identical conditions, so the shift in the predicted interface reflects two separate docking runs rather than re-scoring of a single pose (Figure 3C).

### 3.4. Molecular Interaction of FERM and Sh2 Domain with β-Catenin

**Figure 4:**
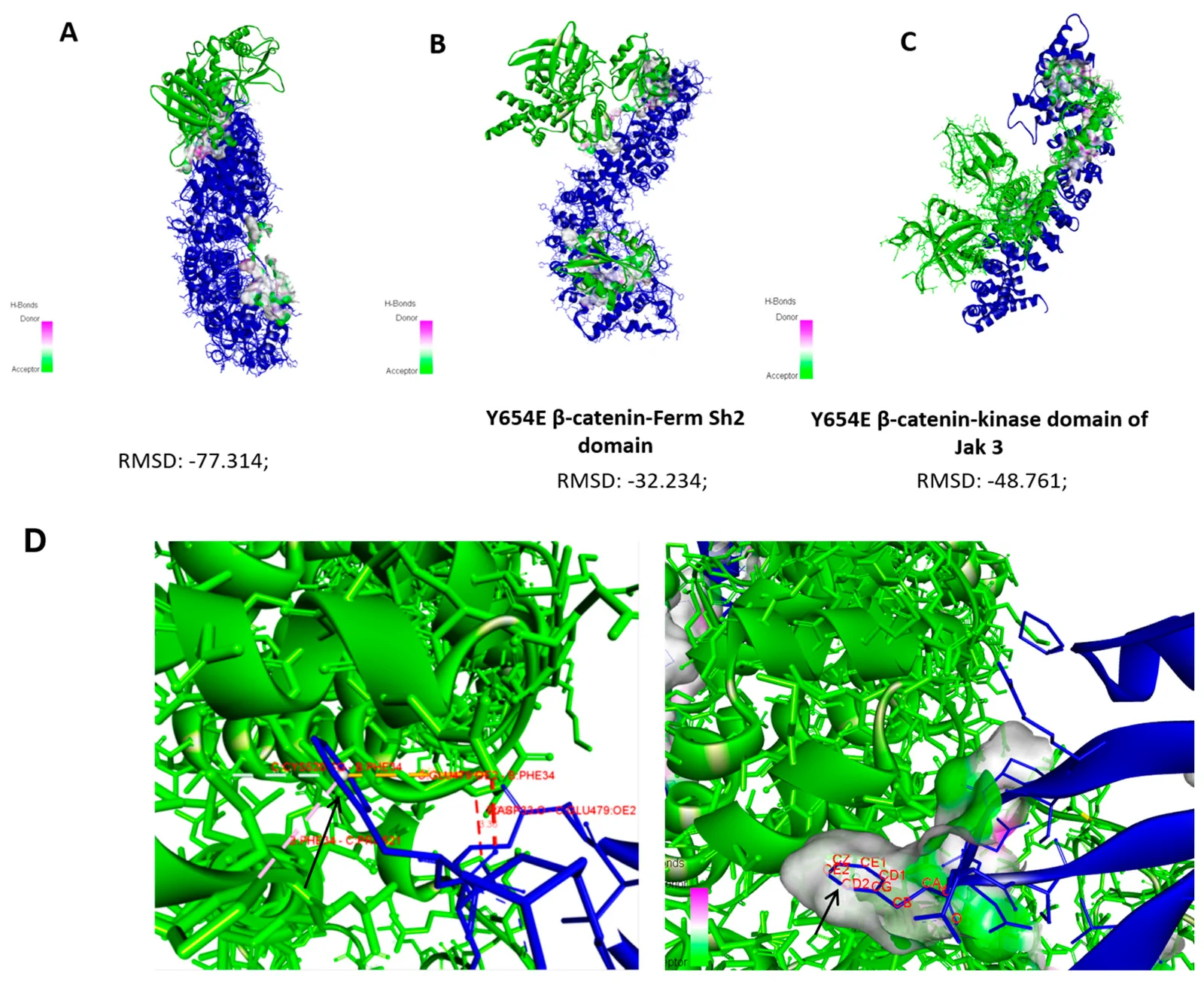
Docking of FERM and FERM_Sh2 with β-Catenin. GRAMMx Protein–Protein Docking server was used to dock both the FERM and FERM_SH2 domains with β-Catenin protein. The figure represents the detailed interactions of the proteins with β-Catenin. (**A**). Y654E β-Catenin docked with the FERM domain of JAK 3. (**B**). Y654E β-Catenin docked with the FERM_SH2 domain. (**C**). Y654E β-Catenin docked with the kinase domain of JAK 3. (**D**). Magnified views of the hydrogen-bonded interface. The values shown beneath panels (**A**–**C**) are the relative interaction scores returned by the docking and superimposition module and are not Cα RMSD values in Å. The hydrogen bonds formed between the FERM domain of JAK 3 and β-Catenin are listed in Table 3.

To assess the impact of structural changes in B-catenin on its association with Jak3 FERM and kinase domains specifically, we constructed the homology models of these domains of Jak3 proteins independently of the full-length protein. These domains were further docked using the GRAMMx Protein–Protein Docking server. Like the full-length Jak 3 protein, the docking of the FERM domain to the full-length wt-β-catenin protein revealed that the binding site is on the armadillo repeats near the C-terminal domain (Figure 4A). However, phosphorylation of tyrosine (Y654) residue of β-catenin induced conformational changes in the structure of the protein, shifting the binding site towards the N-terminal domain of the protein (Figure 4B). The interacting amino acid residues forming hydrogen bonds between the FERM domain and β-catenin protein (Table 3) confirm the shifting of the binding site towards the NTD of the protein, in phosphorylation of Y654 of β-catenin. The scores for these runs are given beneath the corresponding panels of Figure 4; they are the relative interaction scores returned by the docking and superimposition module (dimensionless, more negative values denoting a closer match) and are not Cα RMSD values in Å. The hydrogen bonds formed at the FERM–β-catenin interface are listed in Table 3. Similarly, the FERM-Sh2 domain complex also binds to the ARM repeats of the protein (Figure 4C) but the phosphorylation of Y654 shifts the binding site towards the NTD. The hydrogen bond interactions for the FERM Sh2 domain are represented in Figure 4D.

**Figure 5:**
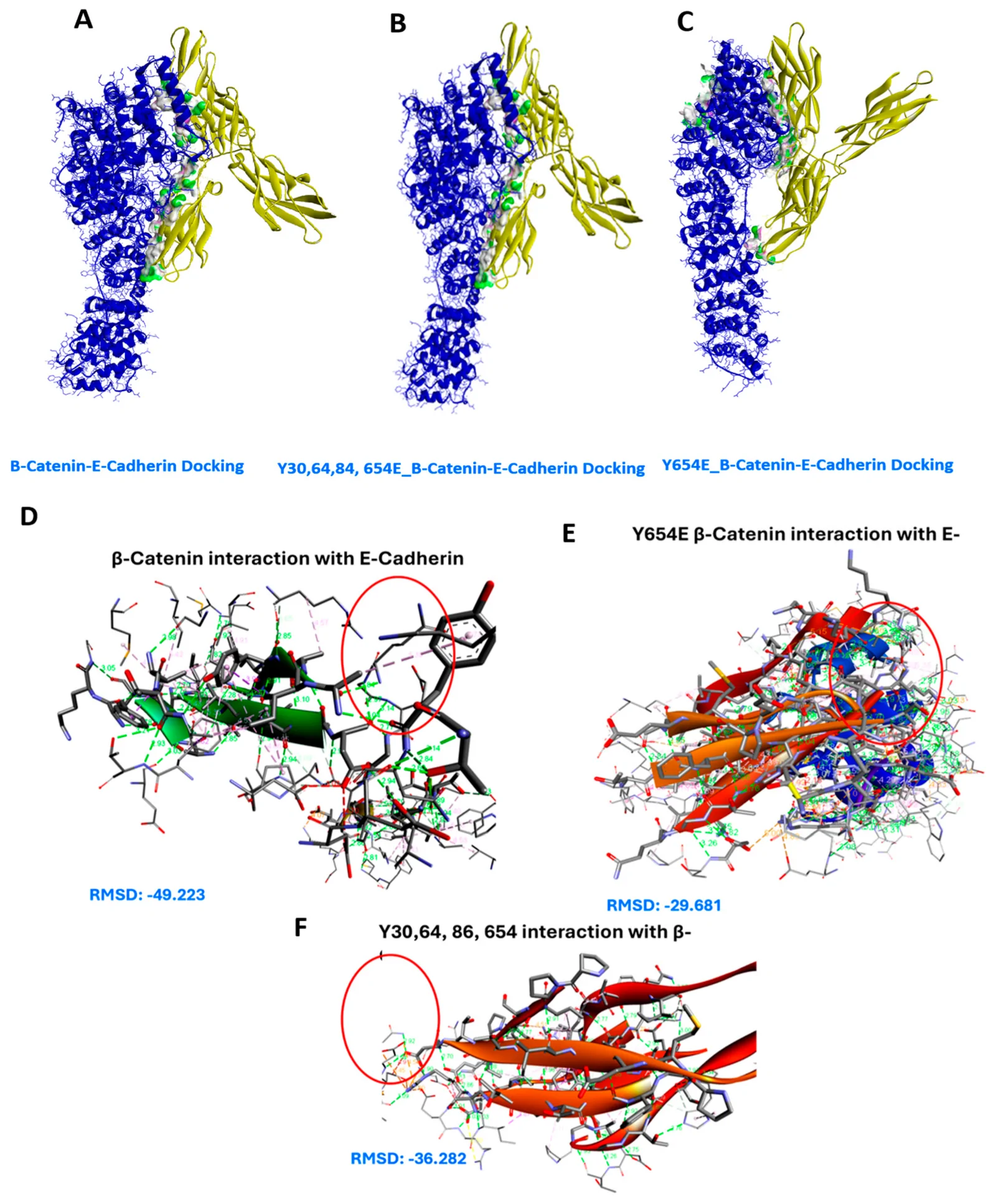
Structural modeling of β-catenin–E-cadherin interactions and the predicted impact of tyrosine phosphorylation. (**Top panel**): Molecular docking models showing the interaction between β-catenin (blue) and E-cadherin (yellow) in the wild-type complex (**left**), the multi-phosphorylated model (Y30, Y64, Y86, and Y654; (**center**)), and the Y654-phosphorylated model (**right**). (**Bottom panel**): Structural superposition of the wild-type and phosphorylated complexes highlighting conformational changes induced by phosphorylation. Red circles indicate regions exhibiting the greatest structural alterations near the β-catenin/E-cadherin binding interface. RMSD values are shown for each structural comparison, demonstrating increasing conformational deviations following phosphorylation, particularly at Y654 and in the multi-phosphorylated model. Panel positions: (**A**) (**top left**), wild-type complex; (**B**) (**top center**), multi-phosphomimetic complex; (**C**) (**top right**), Y654E complex; (**D**) (**bottom left**), wild-type superimposition; (**E**) (**bottom right**), Y654E superimposition; (**F**) (**bottom center**), multi-phosphomimetic superimposition. The values shown beneath panels (**D**–**F**) are the relative alignment scores returned by the superimposition module and are not Cα RMSD values in Å.

To evaluate the effect of tyrosine phosphorylation on the β-catenin–E-cadherin interaction, molecular docking and structural alignment analyses were performed using the wild-type β-catenin structure, a Y654-phosphorylated model, and a multi-phosphorylated model containing Y30, Y64, Y86, and Y654 modifications (Figure 5). Docking analysis demonstrated that phosphorylation altered the structural orientation of the β-catenin–E-cadherin complex. Compared with the wild-type complex, the Y654-phosphorylated β-catenin exhibited a noticeable conformational shift at the E-cadherin binding interface. The multi-phosphorylated β-catenin model (Y30, Y64, Y86, and Y654) showed additional structural rearrangements relative to the wild-type complex (Figure 5, top). Structural superposition analyses further revealed localized conformational changes surrounding the phosphorylated residues and the E-cadherin-binding interface (Figure 5, bottom). These regions, highlighted by red circles, exhibited the greatest deviations between the wild-type and phosphorylated structures. The values returned by the superimposition module were −49.22 for the wild-type complex, −29.68 for the Y654 phosphomimetic complex and −36.28 for the multi-phosphomimetic complex (Figure 5, bottom). These are the dimensionless relative alignment scores reported by the software, not Cα RMSD values in Å; the earlier description of them as RMSD in Å was incorrect and has been corrected, since deviations of that magnitude would not be meaningful for a conventional whole-protein RMSD. Measured against the wild-type value, the Y654 phosphomimetic complex shows the largest departure and the multi-phosphomimetic complex an intermediate one, consistent with partial restoration of the wild-type geometry when Y30, Y64 and Y86 are also substituted. These findings indicate that the substitutions alter the modeled β-catenin–E-cadherin arrangement. Because the docking used the E-cadherin ectodomain rather than the cytoplasmic domain that binds β-catenin in vivo, the comparison should be read as a relative index of conformational change and not as a measure of binding affinity.

## 4. Discussion

The present study provides new structural and mechanistic insights into the regulation of β-catenin by Janus kinase 3 (Jak3) and identifies a previously unrecognized conformational mechanism through which Jak3 preserves adherens junction (AJ) integrity and suppresses epithelial–mesenchymal transition (EMT). By combining homology modeling, molecular dynamics, protein–protein docking, and phosphomimetic analyses, we demonstrate that Jak3-mediated phosphorylation of β-catenin at Y30, Y64, and Y86 restores the native conformation of β-catenin following Src-mediated phosphorylation at Y654, thereby stabilizing its interaction with E-cadherin and maintaining epithelial integrity. These findings extend our previous biochemical observations by providing a structural framework that explains how sequential phosphorylation events regulate β-catenin localization and function.

β-catenin serves dual roles as a structural component of adherens junctions and as a transcriptional co-activator of canonical Wnt signaling. Under physiological conditions, β-catenin associates E-cadherin and α-catenin at the plasma membrane to maintain epithelial architecture. However, disruption of these interactions permits cytoplasmic accumulation and subsequent nuclear translocation of β-catenin, where it activates transcriptional programs associated with proliferation, stemness, invasion, and metastasis. Previous studies have established Src-mediated phosphorylation of Y654 as a critical event that weakens β-catenin interaction with E-cadherin by reducing binding affinity and promoting dissociation from the adherens junction^®^. Our structural simulations provide mechanistic support for these observations by demonstrating that phosphorylation of Y654 induces pronounced conformational rearrangements within the N-terminal domain (NTD), increasing the distance between critical residues and altering the overall structural organization of β-catenin. These conformational changes likely contribute to reduced stability of the E-cadherin/β-catenin complex and facilitate initiation of EMT.

A major finding of this study is that Src-mediated structural remodeling of β-catenin simultaneously creates a favorable interface for Jak3 binding. Docking analyses consistently demonstrated that phosphorylation of Y654 shifts Jak3 interaction from the armadillo repeat region toward the N-terminal domain of β-catenin. Interestingly, both the full-length Jak3 protein and its isolated FERM or FERM–SH2 domains exhibited similar binding preferences following Y654 phosphorylation, suggesting that structural changes in β-catenin rather than intrinsic alterations within Jak3 dictate this interaction. This observation supports a sequential phosphorylation model in which Src activation functions as a priming event that exposes the N-terminal region of β-catenin, thereby permitting recruitment of Jak3 to phosphorylate residues Y30, Y64, and Y86.

The FERM domain is well recognized as the primary mediator of membrane-associated protein interactions within the Jak family. Although structural information regarding Jak3 FERM and SH2 domains has remained limited because of the lack of experimentally solved structures, our homology models demonstrated excellent stereochemical quality and preserved the canonical three-lobed FERM architecture characteristic of JAK family members [25,26]. The modeled FERM–SH2 complex further revealed stable intramolecular interactions suitable for protein docking analyses. These observations are consistent with previous reports demonstrating the importance of the Jak3 FERM domain in mediating interactions with cytoskeletal proteins, including villin, and suggest that the FERM domain serves as a versatile scaffold for regulating epithelial junctional proteins.

An important mechanistic insight emerging from this work is the restorative function of Jak3-mediated phosphorylation. While phosphorylation is generally associated with activation or destabilization of signaling proteins, our simulations indicate that phosphorylation of Y30, Y64, and Y86 reverses the structural distortion induced by Src-mediated Y654 phosphorylation. Restoration of the native N-terminal conformation likely enhances the affinity of β-catenin for E-cadherin and promotes reassembly of adherens junctions. Thus, Jak3 functions not merely as a signaling kinase but as a structural regulator that preserves epithelial architecture by reversing phosphorylation-induced conformational changes. This mechanism provides a molecular explanation for our previous experimental findings demonstrating that Jak3 promotes basolateral localization of β-catenin and enhances epithelial barrier function.

Our phosphomimetic analyses further highlight the differential functional significance of individual tyrosine residues. PROVEAN v1.1.5 predictions identified Y64E and Y86E substitutions as functionally neutral, suggesting that these residues primarily mimic physiological phosphorylation without substantially perturbing protein function. In contrast, Y30E and Y654E substitutions were predicted to be deleterious, indicating that these positions occupy structurally critical locations required for maintaining β-catenin stability. These computational predictions correlate with our docking studies, which identified Y64 and Y86 within the hydrophobic interaction core between Jak3 and β-catenin, whereas Y30 appears to regulate conformational flexibility of the N-terminal region. Together, these observations suggest that phosphorylation of distinct tyrosine residues contributes differently to structural restoration and protein–protein interactions.

The proposed mechanism has important implications for cancer biology. Loss of adherens junction integrity and activation of EMT represent early events in tumor invasion and metastatic dissemination. Aberrant activation of Src kinase and dysregulated β-catenin signaling are common features of colorectal carcinoma and many other epithelial malignancies, where increased nuclear β-catenin activity correlates with poor prognosis. Our findings suggest that Jak3 functions as an endogenous protective regulator that counterbalances Src-mediated disruption of adherens junctions. Rather than promoting oncogenic signaling, Jak3 restores β-catenin conformation and limits its nuclear translocation, thereby maintaining epithelial differentiation and suppressing EMT. These findings are consistent with our previous observations demonstrating that Jak3 deficiency impairs epithelial differentiation and compromises intestinal barrier integrity.

Beyond colorectal cancer, these structural mechanisms may have broader relevance to other epithelial malignancies in which β-catenin dysregulation contributes to metastatic progression, including gastric, pancreatic, hepatocellular, and breast cancers. Because EMT is governed by dynamic interactions between growth factor signaling, Src activation, Wnt signaling, and cell adhesion complexes, modulation of the Jak3–β-catenin axis may represent an important checkpoint integrating these pathways. The structural determinants identified in this study provide a molecular basis for exploring therapeutic strategies aimed at preserving epithelial integrity rather than directly inhibiting β-catenin transcriptional activity, an approach that has historically proven challenging.

Although the computational analyses presented here provide detailed structural insights, several limitations should be acknowledged. First, the three-dimensional models of full-length Jak3 and the FERM–SH2 domains were generated by homology modeling because experimentally resolved structures remain unavailable. Although stereochemical validation indicated high model quality and docking results were consistent across independent simulations, future structural studies using cryo-electron microscopy or X-ray crystallography will be necessary to experimentally validate the predicted interfaces. Second, phosphomimetic substitutions reproduce many but not all biochemical properties of phosphorylated tyrosine residues and therefore cannot completely substitute for direct phosphorylation. Finally, although our structural predictions are strongly supported by previous biochemical studies, additional experimental validation using site-directed mutagenesis, quantitative binding assays, molecular dynamics simulations, and high-resolution structural approaches will further strengthen the proposed sequential phosphorylation model. Several further constraints should be stated explicitly. The analyses presented here are entirely computational: they predict conformational change, but they do not demonstrate phosphorylation-dependent restoration of β-catenin structure, stabilization of the E-cadherin complex or suppression of EMT, each of which requires direct experimental measurement. The β-catenin–α-catenin complex was not modeled, so the statements made about α-catenin binding are inferences drawn from the location of the affected region rather than results of this study. Nuclear translocation of β-catenin was likewise not investigated structurally, and the proposed effect on translocation rests on our earlier cell-based observations rather than on the present models. The E-cadherin docking used the EC1–EC2 ectodomain fragment (pdb id 1q1P); re-docking against the β-catenin/E-cadherin cytoplasmic-domain complex would provide a more direct structural test of the interaction that operates in the adherens junction. Because glutamate substitution alters the residue itself as well as its charge, the conformational effects reported here cannot be separated cleanly from those of genuine phosphorylation; site-specific incorporation of phosphotyrosine, or explicit-solvent molecular dynamics of the phosphorylated species, would be required to make that distinction. Finally, all models were generated with Phyre2, and a comparison with AlphaFold-derived models of Jak3 and β-catenin, together with the interfaces obtained by docking those models, would further strengthen confidence in the conformational changes described here.

## 5. Conclusions

In conclusion, our study establishes a structural mechanism through which Jak3 regulates β-catenin function during maintenance of epithelial homeostasis. Src-mediated phosphorylation of Y654 induces conformational remodeling that facilitates Jak3 recruitment to the β-catenin N-terminal domain, where subsequent phosphorylation of Y30, Y64, and Y86 restores the native protein conformation, stabilizes the E-cadherin/β-catenin complex, and preserves adherens junction integrity. These findings identify Jak3 as a critical structural regulator of epithelial plasticity and reveal a previously unrecognized mechanism linking sequential phosphorylation events to suppression of EMT. The structural insights presented here provide a strong foundation for future development of therapeutic strategies targeting the Jak3–β-catenin signaling axis to prevent tumor invasion and metastatic progression.

## Figures and Tables

**Table 1:** Molecular docking analysis of β-catenin interaction with JAK3. The table summarizes the predicted hydrogen-bond interactions between β-catenin and JAK3 obtained from molecular docking analysis. Four key interacting residue pairs were identified, including TYR432–ASP266, MET1–GLN557, SER448–HIS36, and CYS16–ASP747, with hydrogen-bond distances ranging from 2.39 to 3.01 Å. The spatial coordinates (XYZ) represent the predicted positions of interacting residues within the docked complex. These interactions suggest potential binding interfaces that may contribute to the stability of the β-catenin–JAK3 complex.

Interacting Residues	H-Bond Distance (Å)	Spatial Bond Coordinates (XYZ Coordinates)
TYR 432–Asp 266	2.68	−79.28, 6.26, −52.74
MET 1–GLN 557	3.01	−75.01, 18.74, −83.19
SER 448–HIS 36	2.76	−33.57, −10.77, −74.04
CYS16–Asp 747	2.39	−55.704, 20.62, −81.80

**Table 2:** Y654E β-Catenin docked with Jak3: Hydrogen bond interactions between the ligand and interacting amino acid residues in the target protein binding site. The table summarizes the key interacting residues, hydrogen bond distances (Å), and corresponding spatial bond coordinates (XYZ coordinates) identified through molecular docking analysis. Residues involved in hydrogen bonding interactions include Glu620–Arg57, Glu1041–Lys14, Phe683–Ala94, and Thr679–Phe34, with bond distances ranging from 2.62 to 2.99 Å, indicating favorable ligand–protein interactions.

Interacting Residues	H-Bond Distance (Å)	Spatial Bond Coordinates (XYZ Coordinates)
**Glu 620** **-** **Arg 57**	2.62	−51.05, 11.59, −46.29
**Glu 1041** **-** **Lys 14**	2.90	−55.08, −0.51, −52.129
**Phe 683-** **Ala94**	2.99	−59.06, 12.62, −45.57
**Thr 679-** **Phe 34**	2.96	−65.318, 2.50, −35.21

**Table 3:** Hydrogen bonds formed between the FERM domain of Jak3 and β-Catenin. Interacting residue pairs, hydrogen-bond distances (Å) and spatial bond coordinates (XYZ) obtained for the rank-1 GRAMMx solution. Four hydrogen bonds were identified, with distances between 2.26 and 3.21 Å. These are the interactions referred to in Section 3.4 as confirming the shift of the binding site towards the NTD in phosphorylation of Y654.

Interacting Residues	H-Bond Distance (Å)	Spatial Bond Coordinates (XYZ Coordinates)
Lys 280-Arg 820	2.77	9.29, −31.91, 3.11
His 821-Gly 244	3.21	−9.92, −37.36, −1.83
Pro 862-Glu 358	2.26	−15.95, −23.70, −2.50
Ala 316-Leu 857	3.08	−3.11, −21.48, 1.02

## Data Availability

The original contributions presented in this study are included in the article/Supplementary Materials. Further inquiries can be directed to the corresponding authors.

## Supplementary Materials

The following supporting information can be downloaded at https://www.mdpi.com/article/10.3390/biology15191738/s1, Figure S1: Molecular docking analysis of β-catenin interactions with the JAK3 FERM and SH2 domains; Table S1: Hydrogen bond interactions identified between the FERM domain and β-catenin; Figure S2: Structural model of the FERM domain-β-catenin interaction highlighting key interface residues; Figure S3: Docking models of the β-catenin Y654E mutant (blue) in complex with the FERM domain, SH2 domain, and JAK3 kinase domain (green); Figure S4: Structural effects of individual tyrosine-to-glutamate substitutions in β-catenin and their interactions with JAK3; Figure S5: Predicted interaction interface between β-catenin and JAK3.

## Abbreviations

The following abbreviations are used in this manuscript:

AJ	Adherens Junction
ARM	Armadillo Repeat Domain
CoA	Coenzyme A
EMT	Epithelial–Mesenchymal Transition
FERM	Band 4.1, Ezrin, Radixin, Moesin Homology Domain
FFT	Fast Fourier Transform
JAK3 (Jak3)	Janus Kinase 3
JH1	Janus Homology Domain 1 (Kinase Domain)
JH2	Janus Homology Domain 2 (Pseudokinase Domain)
NTD	N-Terminal Domain
PH	Pleckstrin Homology
PROVEAN	Protein Variation Effect Analyzer
RMSD	Root Mean Square Deviation
SH2	Src Homology 2 Domain
Src	Proto-Oncogene Tyrosine-Protein Kinase Src
WT	Wild Type
Wnt	Wingless/Integrated Signaling Pathway
β-Catenin	Beta-Catenin
pY30	Phosphorylated Tyrosine 30
pY64	Phosphorylated Tyrosine 64
pY86	Phosphorylated Tyrosine 86
pY654	Phosphorylated Tyrosine 654
